# Metabolic potential structures gill symbiont communities in two common shipworm species

**DOI:** 10.1093/ismejo/wrag089

**Published:** 2026-04-23

**Authors:** Ron Flatau, Carson D Bickley, Marvin A Altamia, Mark T Gasser, Daniel L Distel

**Affiliations:** Ocean Genome Legacy Center, Northeastern University, Nahant, MA 01908, United States; Ocean Genome Legacy Center, Northeastern University, Nahant, MA 01908, United States; Philippine Genome Center, University of the Philippines, Diliman, Quezon City 1101, Philippines; Environmental Health and Engineering, Johns Hopkins Applied Physics Laboratory, Laurel, MD 20723, United States; Ocean Genome Legacy Center, Northeastern University, Nahant, MA 01908, United States

**Keywords:** carbohydrate-active enzymes, CAZymes, Cellvibrionacea, lignocellulose degradation, metagenomics, nitrogen fixation, symbiosis, Teredinidae, Teredinibacter, Wood borer, *Lyrodus pedicellatus*, *Teredo bartschi*

## Abstract

Shipworms (Bivalvia: *Teredinidae*) are the most prolific wood consumers in marine environments. These wormlike marine bivalves digest wood using carbohydrate-active enzymes (CAZymes) produced by intracellular bacterial endosymbionts housed within their gills. Although several shipworm species are known to host multiple co-occurring symbiont species, the factors that influence symbiont community assembly, including the phylogenetic identity and metabolic capabilities of the symbionts, remain poorly understood. We sequenced gill symbiont metagenomes from multiple specimens of two shipworm species*, Teredo bartschi* (22 specimens) and *Lyrodus pedicellatus* (14 specimens), which have sympatric distribution in the wild, and which were reared together in laboratory co-culture. From these metagenomes, we assembled 90 metagenome-assembled genomes representing seven distinct symbiont species. The metagenome of each host specimen contained between one and five symbiont species, with each including at least one nitrogen-fixing symbiont. Six of the seven identified symbiont species were found in both host species, demonstrating a lack of host species specificity in these symbioses. We identified patterns of symbiont occurrence and co-occurrence in these two hosts and used these patterns to constrain the core set of CAZyme and nitrogen-fixation gene classes necessary to support host survival. Our results indicate that, in these two host species, symbiont community composition reflects the symbionts’ capabilities for carbohydrate degradation and nitrogen fixation, rather than strict species-specific mechanisms of host and symbiont sorting.

## Graphical Abstract

**Graphical Abstract:**
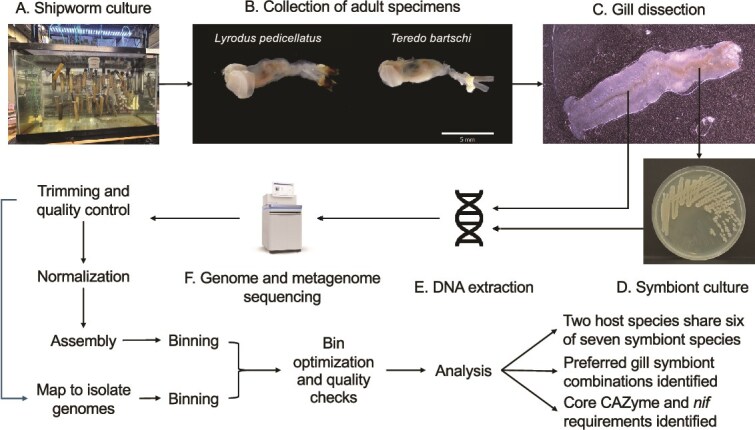

## Introduction

The assembly of microbial communities within eukaryotic hosts is fundamental to shaping both the fitness of individuals and the ecology and evolution of species. However, many eukaryotic microbiomes, such as gut microbiomes, are highly diverse, complex, and variable, making it challenging to isolate and examine the factors that influence their assembly. In contrast, intracellular symbiotic systems, where hosts harbor simple communities of obligate partners, may be too constrained to offer much insight into community assembly processes. Here, we show that symbioses observed in the shipworms *Teredo bartschi* and *Lyrodus pedicellatus* offer an informative balance of complexity, diversity, and specificity, providing opportunities to explore community assembly with unusual precision and statistical power.

Shipworms—wormlike wood-boring marine bivalves of the family Teredinidae—digest wood with the aid of gill-borne intracellular bacterial endosymbionts that produce cellulolytic enzymes [1–3] and fix nitrogen [4], enabling them to survive on a recalcitrant and nitrogen-deficient diet of wood [5]. Often called the “termites of the sea,” shipworms are highly efficient wood consumers. They are found in temperate to tropical waters worldwide at depths from the intertidal zone to more than 150 m [6]. Shipworm larvae (typically 200–300 μm in diameter) settle on and burrow into submerged or floating wood, creating a network of calcium carbonate lined excavations that, although nearly invisible from the surface, can remove the majority of the wood’s internal mass and decimate its structural integrity [6, 7]. This ability to damage submerged wooden structures—including vessels, coastal infrastructure, fishing gear, and aquaculture equipment—causes significant economic losses each year [8–10]. Shipworm damage is also linked to important historical events, such as the untimely termination of Christopher Columbus’s fourth New World expedition [11] and the defeat of the Spanish Armada [12]. However, as the principal consumers of wood in marine environments, shipworms also provide vital ecosystem services [6], such as helping to convert the estimated 300 000 to 70 million cubic meters of large terrestrial wood that enters the marine environment annually [13] into more bioavailable forms, and creating habitat for many other marine and brackish water species [14–16].

Like most other wood-consuming animals, shipworms rely on symbiotic bacteria to aid in wood digestion. However, the shipworm’s symbiont communities are not found within the digestive system [17]. Instead, they are housed intracellularly in a specialized organ within the host’s gills called the gland of Deshayes [18]. These symbionts produce carbohydrate-active enzymes (CAZymes) [19] that are transported from the gill to the gut to aid in wood digestion [1–3, 20, 21].

Although the gill symbionts of all shipworms examined to date fall within a single narrowly circumscribed clade [20, 22–27] within the gammaproteobacterial family *Cellvibrionaceae* [28], the composition and complexity of these communities vary substantially both among host species [20, 21, 25] and among individuals within a given host species [23]. For example, 16S rRNA gene sequence analyses have shown that the gills of the shipworm *L. pedicellatus* harbor multiple closely related, co-occurring symbiont types [27] and that individual specimens of this host species exhibit marked variation in symbiont community composition even when reared together in the same aquarium under identical conditions [23]. Metagenomic analyses showed that similarly complex symbiont communities are observed in the gills of other shipworm species, including *Bankia setacea* [20, 25], *Dicyathifer mannii*, *Bactronophorus thoracites*, *Neoteredo reynei,* and other species of *Bankia* and *Teredo* [21, 25].

Many, but not all, shipworm symbionts can also fix nitrogen [4, 22, 24, 29–31]. Although this ability is thought to help the hosts subsist on their nitrogen-deficient diet of wood [2, 8, 31, 32], it has not been previously determined whether symbiotic nitrogen fixation is essential to host survival. Similar roles in nitrogen metabolism have been proposed for the symbionts of other marine invertebrates, including lucinid clams, stilbonematid nematodes [33], and certain corals and sponges [34].

Symbiotic associations in shipworms appear to be obligate for the hosts as aposymbiotic adults have not been observed. In contrast, host association is not obligate for the symbionts. Many shipworm symbionts can be grown in pure culture on minimal defined media containing only salts, minerals, and a source of reduced carbon—typically a sugar or plant-derived polysaccharide [22, 24, 29]. In the case of non-nitrogen fixers, a source of reduced nitrogen, such as ammonium chloride, is also required [22]. However, no vitamins, amino acids, or host-derived compounds are required for growth.

Although the variability of shipworm symbiont populations has been established, and the potential contributions of symbionts to host metabolism have been proposed, little is known about the factors that influence symbiont community assembly or how the species composition and genomic content of symbiont communities vary within the intact symbioses. To investigate these questions, we compared the composition and complexity of gill symbiont communities of two shipworm species, *T. bartschi* and *L. pedicellatus*, which were reared together in the same experimental aquarium under identical conditions, and that share a sympatric distribution in the wild. We comprehensively characterize the symbiont communities of individual host specimens within these captive populations, producing high-quality metagenome-assembled genomes (MAGs) for seven distinct symbiont species and complete genome sequences for three of these symbiont species that have been brought into pure culture.

## Methods and materials

### Shipworm collection and cultivation

Animals used in this study were collected under Florida Fish and Wildlife Conservation Commission License 36083930 and Special Activity License SAL-25-2733-SR. Naturally occurring submerged wood debris, primarily fallen red mangrove (*Rhizophora mangle*) branches measuring ~15–60 × 2.5–5 cm, was collected by hand at depths of less than 0.5 m from a mangrove thicket in the Indian River Lagoon, Merritt Island, FL (N 28.40605 W 80.66034) on 24–25 January 2020. The collected wood was wrapped in paper towels dampened with water from the site, sealed in loosely fitting plastic bags, and transported by air courier to the Northeastern University Marine Science Center in Nahant, MA. There, the wood was transferred to a glass aquarium containing 50 μm-filtered local Nahant seawater (31 ppt) and maintained at 27°C with aeration. To establish a laboratory breeding colony, larvae produced by shipworms living within this wild-sourced wood were collected on settlement panels constructed of pine molding (12.50 × 1.8 × 1.3 cm). After 7–14 days, settlement panels with newly settled larvae were transferred to a separate aquarium where they were reared to sexual maturity without further contact with wild-caught adult shipworms. Larvae were then collected on fresh settlement panels and the process was repeated serially for five generations, after which multiple generations were allowed to coexist within the colony. Colony maintenance followed protocols described at dx.doi.org/10.17504/protocols.io.e6nvw1qo7lmk/v1.

### Harvesting adult shipworms

**Figure 1:**
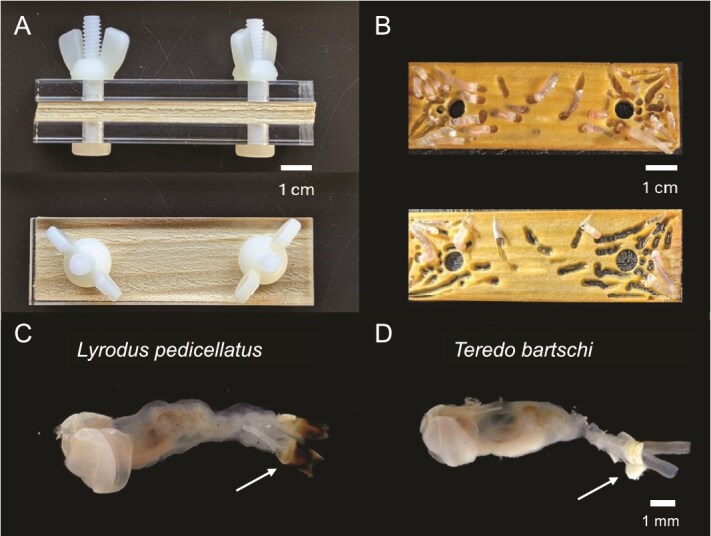
Shipworm culture and harvesting. (A) Laminated settlement panel constructed of stacked 1 mm-thick layers of Scots pine pressed between acrylic plastic sheets and secured with nylon bolts, side view above, top view below. (B) Individual layers of wood after removal from the settlement panel. Upon disassembly of the laminated panels, intact and undamaged adult specimens can be removed from their burrows. (C and D) Mature adult specimens of *Lyrodus pedicellatus* (C) and *Teredo bartschi* (D). Arrows indicate the pallets, which are calcareous structures that shipworms use to seal their burrows against predators and desiccation. The pallets of *L. pedicellatus* are capped by a thick, darkly pigmented proteinaceous periostracum. In contrast, the periostracum of *T. bartschi* is thin and transparent, giving the pallet a whitish appearance.

Thirty-six shipworm specimens were collected from a single laboratory-reared colony. To facilitate the harvesting of intact adult shipworms from wood, larvae were collected and reared to maturity on laminated settlement panels (Fig. 1A). Briefly, 4–5 thin layers of Scots pine (8.25 × 2.54 × 0.1 cm) were sandwiched between strips of clear acrylic plastic (8.25 × 2.54 × 0.635 cm) predrilled with two 0.635 cm holes along the center line, 1.27 cm from either end, and held together with nylon bolts (0.25″, 20 threads per inch). Laminated settlement panels were then placed in laboratory aquaria containing mature breeding adults for 2–3 months, allowing larvae to settle, metamorphose, and grow to maturity. Panels were then carefully disassembled, and individual layers of wood were separated (Fig. 1B), allowing the intact, undamaged mature adult specimens to be gently released from their burrows. The species identity of each collected specimen was then determined based on the morphological features of the pallets. The processing date for each specimen (the date on which that specimen was removed from the colony, dissected, and DNA was extracted) is detailed in Supplementary Table S1. Subsequent metagenomic analyses followed a consistent experimental design and workflow (Supplementary Fig. S1).

### DNA extraction and metagenomic sequencing

Immediately after collection and taxonomic identification, the gills were carefully removed from each shipworm specimen, and any visible larvae in the brood pouches were removed. The gills were then rinsed with sterile seawater and processed for DNA extraction and subsequent metagenomic sequencing. Genomic DNA was extracted from the intact shipworm gills immediately after dissection using a DNeasy Blood and Tissue DNA kit (Qiagen N.V., Hilden, Germany) according to the manufacturer’s instructions. Metagenomic libraries were prepared using a NEBNext Ultra II DNA Library Prep Kit and sequenced with a NovaSeq X Series 10B Reagent Kit (150-bp paired-end) on the NovaSeq 6000 System (Illumina) at the University of Utah High-Throughput Genomics center. See Supplementary Fig. S1 for a diagram of the metagenomic workflow.

### Symbiont isolation and isolate genome sequencing

Symbiont strains SR02026, Lp-A-06, and PMS-3907K.S.1b.02 were isolated from shipworm gills as described in [20], and their genomes were sequenced as in [35]. Briefly, gills were removed by dissection and homogenized in 1.0 ml of shipworm basal medium (SBM) [31] using an autoclave-sterilized glass Dounce homogenizer, as described in [20]. Each homogenate was streaked onto two culture plates. The first contained 1.0% Bacto agar prepared in SBM, adjusted to pH 8.0, and supplemented with 2.0 g/l powdered cellulose (Sigmacell Type 101; Sigma-Aldrich), without a combined nitrogen source. The second contained the same medium supplemented with 0.25 g/l NH_4_Cl. Plates were incubated at 30°C. When individual colonies appeared, they were picked, re-streaked, and regrown on fresh plates, with and without NH_4_Cl supplementation. This process was repeated until clonal isolates were obtained and nitrogen requirements were determined. Genomic DNA was extracted from the resulting clonal isolates, as described in [20], using the Qiagen DNeasy Blood and Tissue Kit following the manufacturer’s recommended protocol for cultured cells, with the exception that DNA was eluted with two 75 μl volumes of AE buffer preheated to 56°C. DNA quality and length were assessed using a TapeStation (Agilent Technologies, US). Sequencing was performed using a MinION (Mk1B) instrument with an R10.4 (FLO-MIN112) flow cell (Oxford Nanopore Technologies, UK) with the Q20+ Chemistry Ligation Sequencing Kit (SQK-LSK112). Base-calling used Guppy v6.4.6 with the high-accuracy algorithm and default read-quality filtering. Adapters were trimmed from reads using Porechop v0.2.4 (https://github.com/rrwick/Porechop) and filtered to remove reads less than 1 Kb using Filtlong v0.2.1 (https://github.com/rrwick/Filtlong). De novo assembly was performed with Flye v2.9.2 (https://github.com/fenderglass/Flye) [36], followed by contig correction and consensus generation with Racon v1.5.0 (https://github.com/lbcb-sci/racon) and Medaka v1.8.0 (https://github.com/nanoporetech/medaka). Assemblies were then circularized using Circlator v1.5.5 https://github.com/sanger-pathogens/circlator [37] and rotated to start at *dnaA* predicted by prodigal v2.6.3 [38]. All analyses used the respective software’s default settings unless otherwise noted. Chromosomal assemblies were produced and annotated using the NCBI Prokaryotic Genome Annotation Pipeline [39].

### Metagenome sequence analysis

The raw metagenome sequence reads obtained from each sample were trimmed, and adaptors were removed using BBDuk (version 39.01, BBTools package). The quality of the trimmed reads was assessed using FastQC. The trimmed reads were normalized using BBnorm (version 39.01, BBTools package) with a target depth of 50× and assembled into contigs using metaSPAdes. Three binning algorithms—Autometa (with the Genome Taxonomy Database; GTDB), MetaBAT 2, and MaxBin2—were employed to group the contigs into bins that approximate distinct bacterial genomes. DASTool was then used to integrate the results of the three binning algorithms and to calculate an optimized, non-redundant set of MAGs that best represented the genomes contained within each metagenome. Additionally, to confirm morphological species assignments, host mitochondrial genome sequences were recovered from each metagenome and were mapped to published mitochondrial genome sequences in Genbank using BBMap (version 39.01, BBTools package) in semi-perfect mode (keeps only alignments with zero mismatches or indels, but ignores any differences where the reference base is “N”).

To improve the detection of low-abundance genomes and the quality of their associated MAGs, the original trimmed and quality-controlled raw reads (without normalization) from each sample were mapped to a set of 82 previously assembled isolate genomes and MAGs (FASTA file and checkM results are provided in the supplementary data file, Supplementary_data_file.zip, http://dx.doi.org/10.5061/dryad.ksn02v7jd). Mapping was performed using BBMap (version 39.01, BBTools package) in semi-perfect mode. The mapped reads for each DASTool-optimized MAG were then assembled using SPAdes, and contigs were binned and optimized as described above. Redundant bins (those representing the same symbiont genome) were identified using FastANI. When redundant bins were identified, the bin of best quality was determined using CheckM [40] quality criteria (e.g. completeness, contamination, heterogeneity), and was retained for further analysis.

### Gene annotation

Genome features for each MAG and isolate were annotated with the NCBI Prokaryotic Genome Annotation Pipeline 2024-07-18.build7555 [41] with the flag -S “Bacterium sp.” CAZyme annotations were further refined, and substrate predictions were made using dbCAN3 v5 [42], which integrates HMMER, DIAMOND, and Hotpep predictions; only genes supported by at least two of the three methods were retained as high-confidence CAZymes. Subfamily designations were assigned to high-confidence CAZyme calls using dbCAN_sub, which relies on HMM-based models. All substrate predictions and comparative analyses of CAZyme diversity were conducted at the subfamily level and therefore considered only confidently annotated modules.

### Symbiont species delimitation

Species represented by each symbiont genome and MAG were differentiated by genomic average nucleotide identity (gANI) using FastANI [43], with species delimitation cutoffs of gANI >95% and alignment fraction (AF) > 60% [43, 44].

### Phylogenetic analysis of symbiont genomes and MAGS

For each symbiont isolate genome and MAG, the Genome Taxonomy Data Base Toolkit (GTDB-Tk; [45, 46]) was used to identify 120 single-copy marker gene regions and to generate multiple sequence alignments of concatenated amino acid sequences for phylogenetic inference. A maximum likelihood tree was then constructed from the alignment using the IQ-TREE web server [47] with default settings, which included ModelFinder [48] for model selection and Ultrafast Bootstrap [49] for support estimation with 1000 bootstrap replicates.

### Relative abundance of symbionts within individual specimens

Trimmed, but not normalized, metagenomic reads were mapped to MAGs using the BBTools package of BBMap (version 39.01). For each host specimen, the total number of mapped reads across all MAGs was calculated, and the relative abundance of each MAG was computed using the formula: 


$$ \begin{array}{l}\displaystyle \frac{\#\ \mathrm{of}\ \mathrm{reads}\ \mathrm{in}\ \mathrm{the}\ \mathrm{given}\ \mathrm{MAG}}{\mathrm{total}\ \#\ \mathrm{of}\ \mathrm{MAG}\ \mathrm{reads}\ \mathrm{in}\ \mathrm{the}\ \mathrm{given}\ \mathrm{metagenome}} \ast 100 = \%\ \mathrm{abundance}\ \mathrm{per}\ \mathrm{MAG} \end{array} $$

### Statistical analyses of symbiont abundance, prevalence, and co-occurrence

To assess the relationship between symbiont prevalence and abundance, we calculated correlation coefficients using both Pearson’s product–moment correlation [50] and Spearman’s rank correlation [51]. For each host species (*L. pedicellatus* and *T. bartschi*) and for the combined dataset, we computed the average percent abundance per specimen of each symbiont species, applying zero values when a given symbiont was absent. The percent prevalence was defined as the proportion of host individuals harboring a given symbiont. Pearson’s *r* was used to evaluate linear associations, whereas Spearman’s ρ provided a nonparametric test of monotonic association based on ranked values, which is more robust to small sample sizes and non-normal data distributions [52]. All analyses were performed in Python (v3.11) using the scipy.stats.pearsonr and scipy.stats.spearmanr functions, with significance assessed at α = 0.05.

To test whether the distribution of symbiont species among host specimens differed from random acquisition, we analyzed the presence–absence patterns of seven symbiont taxa among the examined host species. Patterns of symbiont community assembly were then evaluated using a richness-preserving Monte Carlo randomization test. For each host, the observed number of symbiont species was held constant while species identities were randomized from the pool of seven candidates, thereby preserving host-level richness but removing symbiont-species-specific associations. This randomization was repeated 10 000 times to generate null frequency distributions for all symbiont combinations. Observed frequencies were then compared to null expectations, and two-sided *P* values were calculated.

To account for multiple testing across combinations, we applied both the Benjamini–Hochberg false discovery rate (FDR) procedure [53] and Holm’s sequential Bonferroni method [54]. Combinations were considered significantly over- or under-represented when *q* < 0.05 (FDR) or *P* < .05 (Holm).

Unobserved co-occurrences (pairs of symbionts that were never detected together in any host) were evaluated using an exact Poisson–binomial test [55]. The null probability of observing a specific pair for each host with richness k_i_ was calculated as (5k_i_−2)/(7k_i_) for k_i_ ≥ 2, and zero for k_i_ < 2. The probability of observing no co-occurrences across the dataset was then ∏_i_(1 − p_i_), providing an exact *P* value for complete absence under the null. These values were adjusted for multiple comparisons using FDR and Holm corrections as above.

### Lignocellulose-active CAZyme diversity

We used dbCAN3 v5 [42] to identify lignocellulose-active CAZymes within the symbiont genomes and metagenomes and to predict their activities and substrates. We then identified the minimal or core set of lignocellulose-active CAZymes common to all metagenomes. Next, we examined differences in lignocellulose-active CAZyme subfamily diversity among hosts grouped by symbiont community richness, defined as the number of distinct symbiont species detected in each host specimen. Lignocellulose-active CAZyme diversity was defined as the number of unique lignocellulose-active CAZyme subfamilies detected in each symbiont MAG. Lignocellulose-active CAZymes were defined as those that target lignocellulose-associated structural polymers and the oligomers associated with their degradation. For the overall group comparison, we employed a Kruskal–Wallis test. Where that test revealed a significant difference among groups (*P* < .05), we conducted post hoc pairwise comparisons using Dunn’s test with Holm correction. All analyses were performed in R, utilizing rstatix and rcompanion for hypothesis testing.

## Results and discussion

### Identification of host species

Among the 36 specimens examined, two shipworm species, *L. pedicellatus* (14 specimens) and *T. bartschi* (22 specimens), were identified based on morphological characters of the pallets. The pallets of the former species bear thick, darkly pigmented periostracal caps, whereas those of the latter are thin and transparent, revealing the white calcareous core of the pallets (Figs. 1C and D). Mitochondrial genomes recovered from each host specimen supported morphological species identifications. The complete mitochondrial genomes and mitochondrial cytochrome oxidase subunit 1 (*COI*) genes of specimens identified as *L. pedicellatus* and *T. bartschi* in this study matched their respective reference sequences in GenBank (OM910820 and OM910823) with >97% (complete mitogenome) and >99% (*COI*) nucleotide sequence identity, respectively (Supplementary Table S2). In contrast, in between-species comparisons, the mitogenomes and *COI* genes of *L. pedicellatus* and *T. bartschi* shared <92% nucleotide sequence identity, consistent with differences observed among other shipworm species [56].

### Symbiont species delimitation and classification

**Figure 2:**
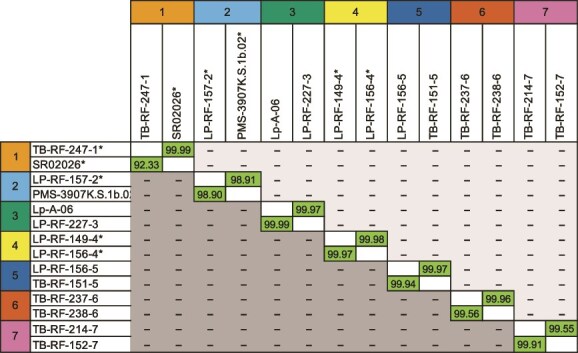
Symbiont species delimitation based on gANI and AF. This similarity matrix displays gANI (above the diagonal) and AF values (below the diagonal) for a subset of MAGs and isolate genomes used in this study. Two representative sequences are presented for each of the seven symbiont groups identified in co-cultured specimens of the shipworms *Lyrodus pedicellatus* and *Teredo bartschi.* For symbiont groups that have been isolated in pure culture, both the isolate’s genome sequence (indicated by asterisks) and a representative high-quality MAG are shown. For the remaining symbiont groups, two representative high-quality MAGs are displayed. For all comparisons, within-group gANI and AF values exceed 98% and 92%, respectively, surpassing the threshold values (96.5% and 60%) proposed for delimiting bacterial species [44]. In contrast, between-group values are too low to be calculated using FastANI [43] and are represented by dashes. These results support the designation of symbiont groups 1–7 as distinct bacterial species. For a comprehensive gANI comparison of all isolates and MAGs used in this study, see Supplementary_data_file.zip (http://dx.doi.org/10.5061/dryad.ksn02v7jd).

Eighty-six high-quality MAGs (>90% completeness, <0.5% contamination) were recovered from the metagenomes of the 36 shipworm specimens (Supplementary_data_file.zip, http://dx.doi.org/10.5061/dryad.ksn02v7jd). The average read depth and total Gb generated per sample were 70.5× and 300 457 047, respectively. The observed average completeness was 98.9% ± 1.5%. An additional four MAGs, with completeness ranging from 87% to 90% and contamination ranging from 5% to 10%, were also determined to be suitable for phylogenetic analysis and were therefore included in this study [40, 57]. The 86 MAGS were classified into 7 similarity groups based on gANI comparisons (>98% gANI, >64% AF within groups, <80% gANI between groups; Fig. 2). These differences substantially exceed values (<96.5% gANI, and >60% AF) previously suggested as thresholds for bacterial species delimitation [44]. For this reason, we hereafter refer to these groups as symbiont species 1–7.

The MAGs identified as species 1 and the symbiont isolate designated as SR02026 match at the species-level (>98% gANI, >98% AF) [29, 58, 59] with *Teredinibacter turnerae* str. T7901 (Genbank NZ_CP149819.1; ATCC 398, a previously cultured symbiont that occurs widely among shipworm species [29]. Additionally, species 2 showed a species-level match to *Cellvibrionaceae* bacterium PMS-3907K.S.1b.02 (GenBank SAMN51758144), isolated from a specimen of the shipworm *Tamilokus mabinia,* collected in February 2018 in Balayan Bay, Mabini, Batangas, Philippines. Similarly, symbiont species 3 showed a species-level match to *Cellvibrionaceae* bacterium Lp-A-06 (GenBank SAMN50737058) isolated on 14 June 2018 from a specimen of *L. pedicellatus* obtained from a culture propagated from specimens collected in Alamitos Bay, CA, USA in 1979.

### Phylogenetic analyses of symbiont genomes and MAGs

**Figure 3:**
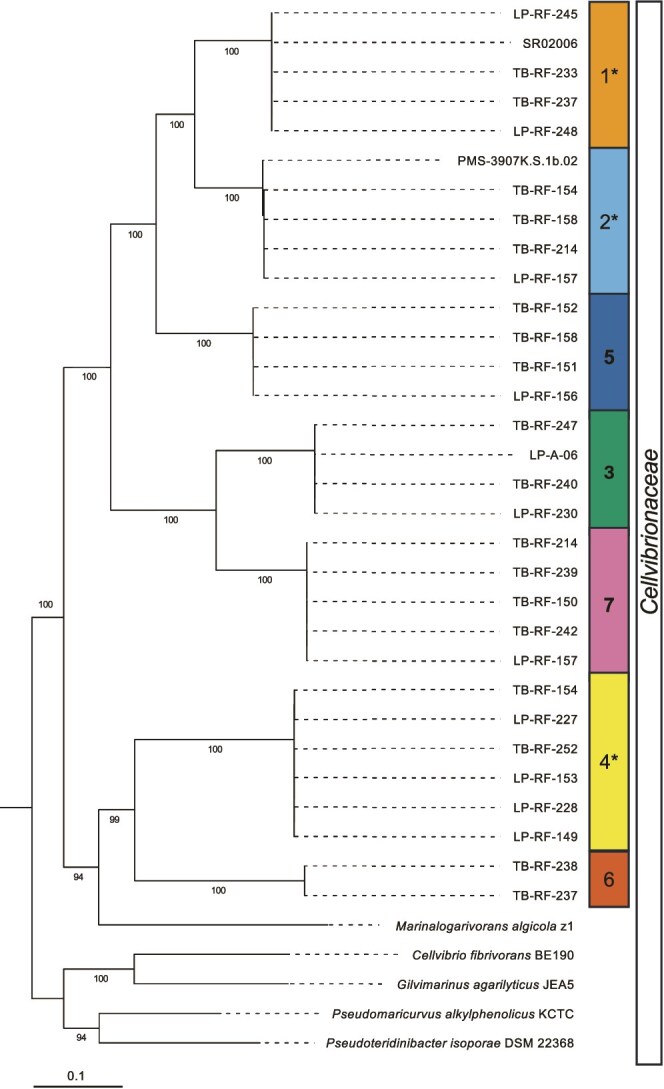
Phylogenetic relationships among symbiont MAGs, isolates, and reference representatives of *Cellvibrionacea*. A maximum likelihood tree was generated using IQtree [47] based on an alignment of 120 conserved marker gene regions identified using the GTDB-Tk from representative MAGs and isolate genomes used in this study. Isolate genomes are in bold. Symbiont species are numbered from 1 to 7. Asterisks indicate species capable of nitrogen fixation. Bootstrap support values, expressed as a percentage of 1000 replicates, are shown at each node. Scale bar indicates length representing 0.1 expected substitutions per site.

Phylogenetic analyses based on 120 conserved marker genes selected using the GTDB-Tk (Fig. 3) demonstrate that all seven symbiont species fall within the gammaproteobacterial family *Cellvibrionaceae*, a bacterial group that is primarily found in marine or saline environments and that contains many complex polysaccharide degraders [28], including previously identified shipworm symbiont species [22, 24].

### Host-symbiont species specificity

Our data provide no evidence of host species-specificity among these seven symbiont species. Six of the seven symbiont species (1–5 and 7) were detected in the metagenomes of both host species, whereas one symbiont, species 6, was found only in *T. bartschi.* The latter symbiont was observed in just two individuals and only at low abundance (<3%). Therefore, its absence from the *L. pedicellatus* metagenomes, of which fewer individuals were sampled*,* likely reflects insufficient sampling of a rare symbiont species rather than evidence of a species-specific association with *T. bartschi*.

### Variation of symbiont community species composition

**Figure 4:**
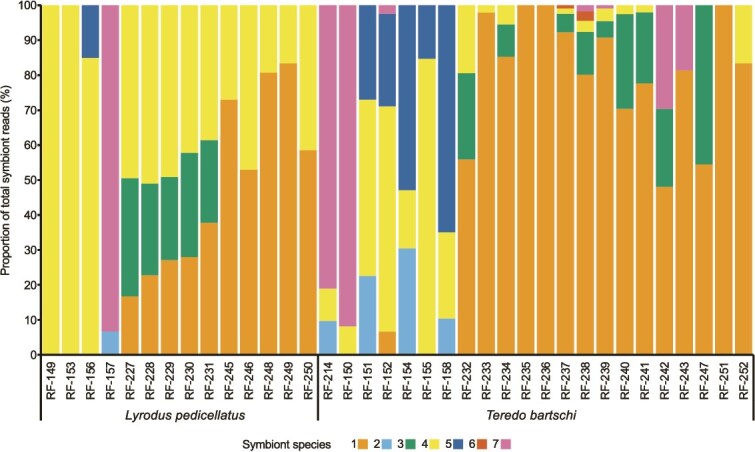
Symbiont community compositions of 36 shipworm specimens. A stacked bar chart showing the symbiont community composition of 22 individual specimens of *Teredo bartschi* and 14 individual specimens of *Lyrodus pedicellatus,* as estimated by the percentage of total symbiont reads in each gill metagenome assigned to each symbiont MAG. Host specimen IDs are listed below each column. Orange, symbiont species 1; light blue, symbiont species 2; green, symbiont species 3; yellow, symbiont species 4; dark blue, symbiont species 5; red, symbiont species 6; and magenta, symbiont species 7. Species 1–3 are conspecific with *Teredinibacter turnerae* T7901, *Cellvibrionacea* bacterium PMS-3907K.s.1b.02, and *Cellvibrionacea* bacterium Lp-A-06, respectively.

As previously reported for *L. pedicellatus* based on 16S rRNA gene analyses [23, 27], symbiont community composition determined by metagenomic analysis varied substantially among individual specimens of *L. pedicellatus* and *T. bartschi* with respect to symbiont species identity and relative abundance, even when grown together under identical conditions in the same experimental aquarium. Among different hosts, the abundance of individual symbiont species ranged from 0% to 100% (Fig. 4). None of the seven identified symbiont species was detected in all host specimens.

### Symbiont species richness

Of the two host species examined, *T. bartschi* displayed the more species-rich gill metagenomes with up to five symbiont species per host individual compared to a maximum of three per individual in *L. pedicellatus*. *Teredo bartschi* also averaged slightly, but not significantly, more symbiont species per individual, with an average of 2.7 ± 1.0 symbionts per individual as compared to 2.2 ± 0.7 for *L. pedicellatus.*

**Figure 5:**
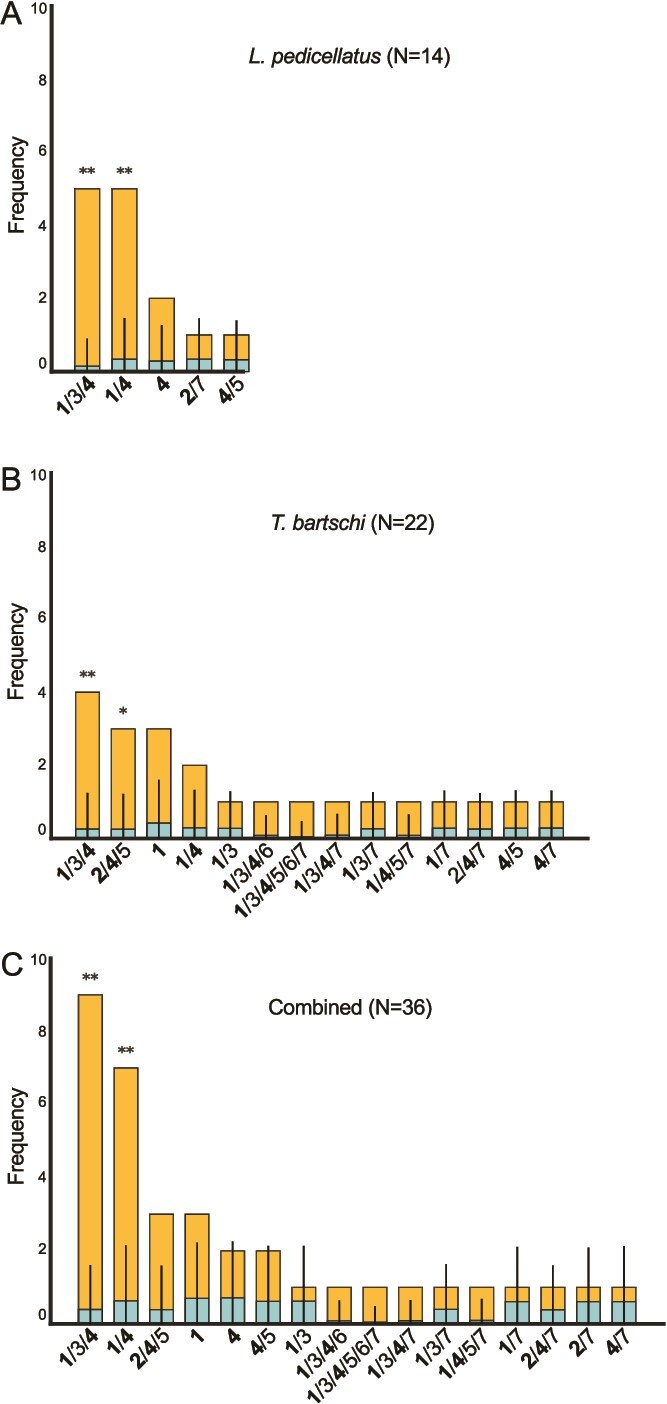
Co-occurrence patterns of seven symbiont species in individual specimens of *Lyrodus pedicellatus* and *Teredo bartschi.* Bar plot showing the number of host specimens exhibiting each of 16 observed patterns of symbiont co-occurrence within the gill symbiont communities of (A) 14 individual specimens of *L. pedicellatus*, and (B) 22 individual specimens of *T. bartschi*. The combined totals for both species are shown in (C). The frequencies of all observed patterns of symbiont species occurrence and co-occurrence are listed in order from most to least frequent (left to right). Nitrogen-fixing symbiont species are labeled in bold type. Observed frequency data are in orange. Blue indicates the frequencies expected under a null model, assuming a random assortment of symbionts where each symbiont species has an equal likelihood of selection and the observed symbiont richness per specimen is preserved. Whiskers denote the 95% confidence envelopes of the null distribution. Asterisks mark combinations occurring significantly more frequently than expected after multiple-test correction (Benjamini–Hochberg FDR q-values and Holm-adjusted *P* values). * = FDR q and Holm *P* ≤ .05 and ** = FDR q and Holm *P* ≤ .01.

Although most individuals of both host species harbored more than one symbiont, in a few host individuals, only one symbiont species was detected. Among the seven symbiont species, however, only two symbiont species (1 and 4) were observed to occur as the sole member of a single host’s gill metagenome (Fig. 5). Symbiont species 1 occurred as the sole symbiont only in *T. bartschi* (three individuals), whereas symbiont species 4 occurred as the sole symbiont only in *L. pedicellatus* (two individuals). These results indicate that the genomes of species 1 and species 4 each encode all genes necessary to support their respective hosts’ survival.

Species 1 and 4 were also the most prevalent in both host species. Symbiont species 1 and 4 were present in 93% and 72% of *L. pedicellatus* specimens, respectively, and both occurred with equal prevalence (73%) in *T. bartschi* specimens. Additionally, symbionts 1 and 4 were the two most abundant symbiont species in both host species. Symbiont species 1 accounted for an average of 56% of symbiont reads in *T. bartschi* and 34% of reads in *L. pedicellatus.* Symbiont species 4 accounted for 14% of symbiont reads in *T. bartschi* and 48% of reads in *L. pedicellatus.*

### Symbiont prevalence vs. abundance

**Figure 6:**
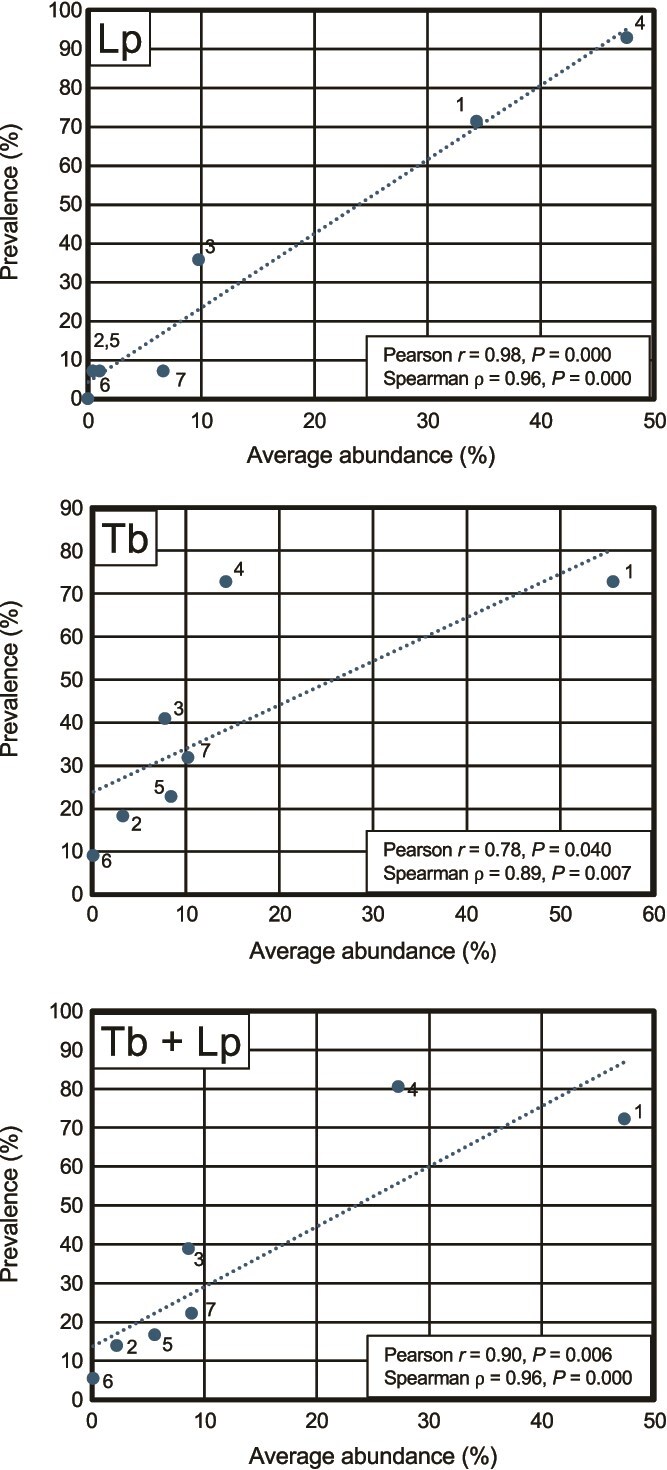
Relationship between symbiont prevalence and average abundance across host specimens. Scatter plots show the correlation between average percent abundance (fraction of the total symbiont community accounted for by a single symbiont) and percent prevalence (fraction of host individuals in which each symbiont was detected) for *Lyrodus pedicellatus* (left; 14 specimens), *Teredo bartschi* (center; 22 specimens), and the combined dataset (right; 36 specimens). Each point represents one of the seven symbiont species and is labeled with its species number. Dashed lines indicate least-squares linear regressions. Annotations report Pearson’s correlation coefficient (*r*) and Spearman’s rank correlation coefficient (ρ) with associated *P* values. Strong positive correlations indicate that symbionts most frequently encountered across hosts are also those comprising the greatest proportion of the overall community.

Symbiont prevalence (the proportion of host specimens in which each symbiont species was detected) and relative abundance (the proportion of the symbiont community in a given host specimen accounted for by a given symbiont species) showed a strong and significant positive correlation in both host species and in the combined set (Pearson *r* = 0.90, *P* = .006; Spearman ρ = 0.96, *P* < .001) when symbiont abundance was averaged across all specimens, and zero values were assigned when a given symbiont was not detected (Fig. 6). These results demonstrate that symbiont species that proliferate more strongly within these host species are more likely to be acquired by these host species, and vice versa. Because symbiosis is obligate for the host (i.e. hosts cannot reach sexual maturity without acquiring one or more symbionts), this correlation suggests an alignment between host fitness (the ability to survive and reproduce) and symbiont fitness (the ability to be acquired by and to proliferate within the host).

### Co-occurrence of symbiont species within individual metagenomes

Within the metagenomes of individual specimens from the two host species, a total of 16 unique patterns of symbiont co-occurrence were observed. These include patterns with 1, 2, 3, 4, and 5 of the 7 detected symbiont species per host individual (Fig. 5). Six of the seven identified symbiont species were observed in *L. pedicellatus*, with a total of five patterns of occurrence. All seven symbiont species were observed in *T. bartschi*, with a total of 14 unique occurrence patterns. Three unique combinations (1,4; 1,3,4; and 4,5) were observed in both host species.

**Figure 7:**
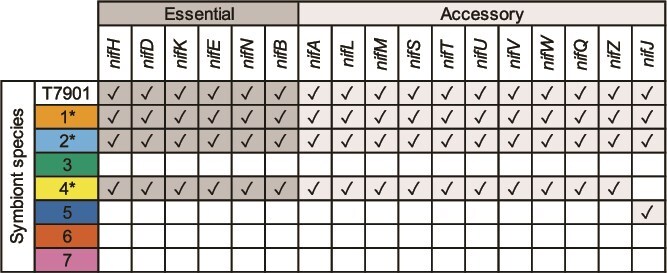
Presence–absence of nitrogen-fixation (*nif*) genes in the genomes of seven symbionts found in the shipworms *Lyrodus pedicellatus* and *Teredo bartschi.* Seventeen genes—including the essential core nitrogen fixation genes, *nifH, D, K, E, N*, and *B*—are present in species 1, 2, and 4, predicting their nitrogen-fixing capability. Species 1 and 2 encode the additional accessory protein NifJ. In contrast, the remaining symbiont species lack all *nif* gene classes, except for species 5, which encodes the accessory protein, NifJ. Checkmarks indicate the presence of each *nif* gene class. Essential *nif* genes are shaded in dark grey; accessory *nif* genes are shaded in light grey. Asterisks indicate the predicted ability to fix nitrogen. For comparison, the *nif* genes present in the genome of *Teredinibacter turnerae* T7901, a strain that has been demonstrated to fix nitrogen, are presented.

We performed Monte Carlo randomization tests to compare the frequency of each observed symbiont combination with its expected frequencies under a null model that assumes a random association of hosts and symbionts but preserves the species richness per specimen. The results showed that the symbiont species triplet 1,3,4 (*P* < .01) and the pair 1,4 (*P* ≤ .01) were significantly more prevalent than the random expectation for *L. pedicellatus*. In *T. bartschi*, the triplets 1,3,4 and 2,4,5 were significantly enriched (*P* < .01) over the null model (Fig. 7). Some symbiont pairs (1–2, 2–3, 2–6, 3–5, and 5–6) were never observed across all hosts. To evaluate whether these absences reflect biological constraints or stochastic variation, we applied exact Poisson–binomial tests under the null model that preserves richness. After adjusting for multiple testing, the absence of these species pairs in the observed data did not differ significantly from expectations under the null model. Taken together, these data suggest that complementary interactions among symbionts or between symbionts and hosts likely influence the assembly of these communities, whereas competitive or antagonistic interactions, though possible, are not statistically supported.

### Symbiont nitrogen fixation

Symbiotic nitrogen-fixation has been shown to occur in shipworm gill tissues and in symbiont isolates and is proposed to contribute to the host’s nitrogen needs [4, 29–31, 60]. Here, we show that three of the seven identified symbiont species (1, 2, and 4) are predicted to fix nitrogen. The genomes of these species encode all the essential core genes, *nifH, D, K, E, N,* and *B*, that are proposed to predict nitrogen-fixing capability, as well as the regulatory genes *nifA, L,* and the accessory genes *nifM, S, T, U, V, W, Q,* and *Z* [61] (Fig. 7). *NifJ* is also present in species 1 and 2, but is absent in species 4.

As further evidence of their nitrogen-fixing capability, two of these three symbiont species (1 and 2) have been cultivated *in vitro* and grow well on plates and in liquid media with no added source of combined nitrogen. Additionally, all three share a nearly identical complement of *nif* genes as found in the genome of *T. turnerae* T7901 [59], which has been empirically demonstrated to fix nitrogen [4, 30].

In contrast to species 1, 2, and 4, which bear a full complement of *nif* genes, the remaining symbiont species lack all *nif* gene classes except *nifJ,* which is present only in species 5. This gene exhibits variable distribution among nitrogen-fixing bacteria [61] and is present in the genomes of bacteria that do not fix nitrogen, such as *Yersinia enterocolitica* and *Yersinia pseudotuberculosis* [62, 63], indicating that it is not essential for, and does not predict, nitrogen fixation. Thus, symbionts 3, 5, 6, and 7 lack the gene classes needed for nitrogen fixation.

We observed that all examined symbiont metagenomes included at least one of the three symbiont species predicted to fix nitrogen, and nearly half (17 of 36) included two. Given a null model where nitrogen fixation does not influence symbiont selection and species richness values are preserved, the likelihood of observing at least one nitrogen-fixing symbiont species in each of the 36 metagenomes is <3 × 10^−5^. This result strongly suggests that symbiotic nitrogen fixation is highly advantageous, if not essential, to host survival.

### Constraining the core *nif* gene classes central to host survival

By comparing the genomes of the three nitrogen-fixing symbiont species, we can constrain the core genes required for nitrogen fixation in these symbiotic systems. Symbiont species 1, 2, and 4 encode 17 *nif* gene classes, 16 of which are shared among all three symbiont genomes, suggesting that these 16 classes represent an upper limit on the core or minimum set of *nif* genes necessary to support host survival.

### Constraining the core CAZymes central to host survival

All shipworm symbiont gill microbiomes in this study, as well as those examined in other wood-boring shipworm species [1, 3, 20], contain symbionts with genomes rich in lignocellulose-degrading CAZymes, which are proposed to support wood digestion by the hosts. By comparing the CAZyme gene content of the symbiont communities observed in *L. pedicellatus* and *T. turnerae*, we can begin to constrain the minimal set of CAZymes required by these hosts to survive on their wood-based diet.

Because symbiont species 1 and 4 were each observed to occur as the sole member of the symbiont community in multiple host specimens, either of these symbiont species, by itself, must provide all symbiont-dependent functions required for the survival of the hosts. Similarly, the combination of symbiont species 2 and 7 must also include all genes necessary for host survival. Thus, the set of lignocellulose-degrading CAZymes shared by symbiont species 1, 4, and the combination of 2 and 7, represents an upper limit on the core or minimum set of lignocellulose-degrading CAZymes needed for host survival.

The CAZy database (www.cazy.org) categorizes CAZymes into six classes and over 500 families, based on sequence homology [64]. These families are further classified into numerous subfamilies presumed to share the same activities and substrates [42, 65]. Because CAZyme proteins often contain multiple independently acting catalytic and binding modules selected from multiple families and subfamilies, it is most practical to explore CAZyme diversity and function based on the classification and enumeration of modules rather than proteins.

Using the tools available in dbCAN3 v5, [42] we identified genes encoding 124 CAZyme families and 444 unique CAZyme subfamilies in the genomes of symbiont species 1 through 7. We then identified a subset of 58 CAZyme module subfamilies shared by the genomes of symbiont species 1, 4, and the combination of 2 and 7. These constitute the core CAZyme subfamilies shared by all symbiont communities. Next, we used the substrate prediction tool in dbCAN3 v.5 to identify 23 module subfamilies within this core set that are predicted to act on lignocellulose components (cellulose, hemicellulose, and lignin) or the oligosaccharides produced by the deconstruction of these components. Hypothetically, these include, but are not limited to, the minimal core set of CAZyme activities and structures required for lignocellulose degradation by *T. bartschi* and *L. pedicellatus.*

The identified core set of 58 CAZymes predicts activities targeting a wide variety of bonds within all three major components of wood, including cellulose, hemicellulose, and lignin (Supplementary Table S3). These predictions encompass 17 distinct EC activities, including glycoside hydrolases, carbohydrate esterases, and oxidoreductases potentially acting on cellulose, xylan, mannan, β-glucan, galactan, arabinan, xyloglucan, and lignin. These include both endo- and exo-acting glycosidases (EC 3.2.1.x) for depolymerizing diverse plant polysaccharide backbones, as well as accessory enzymes such as licheninase (EC 3.2.1.73) and arabinofuranosidase (EC 3.2.1.55) for debranching hemicellulose, and acetylxylan esterase (EC 3.1.1.72) for deacetylating xylans and xylo-oligosaccharides. Additionally, lignin-modifying peroxidases potentially improve enzyme access to the carbohydrate components of lignocellulose. Finally, five distinct CBM subfamilies potentially facilitate enzyme binding to cellulose, β-glucans, xylans, mannans, and galactans, increasing enzyme access to the carbohydrate matrix. This enzyme repertoire suggests the coordinated breakdown of lignin, cellulose, and hemicelluloses, indicating a community well-adapted for lignocellulose degradation. Thus, the composition of this core CAZyme module subfamily set strongly indicates that complete lignocellulose degradation, rather than dependency on any single component of wood, is an essential feature of shipworm gill microbiomes, and is therefore a potentially important determinant of gill symbiont community composition.

### Lignocellulose-active CAZyme content as a function of community size

**Figure 8:**
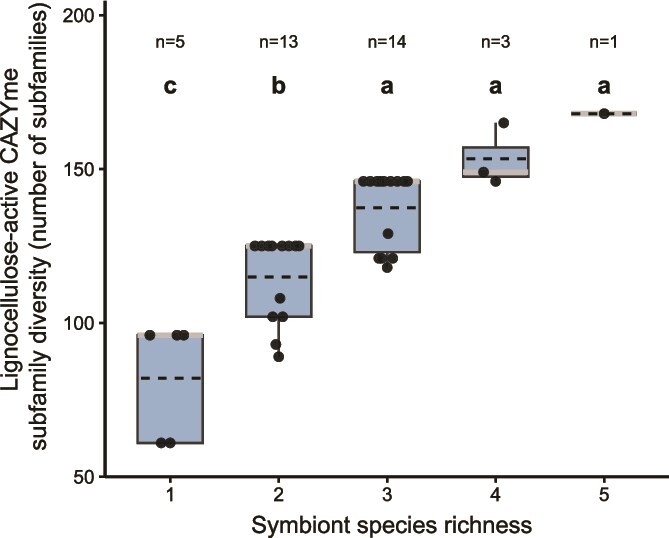
Lignocellulose-active CAZyme subfamily diversity versus symbiont community richness. Box plot demonstrating the relationships between lignocellulose-active CAZyme subfamily diversity and symbiont species richness among gill metagenomes of 36 individual specimens of *Lyrodus pedicellatus* and *Teredo bartschi*. CAZyme diversity increases significantly (Kruskal–Wallis test, α < 0.05) as symbiont richness rises from one to three symbiont species per community (Dunn’s test with Holm correction, α < 0.05). Boxes, interquartile range (Q1–Q3); grey horizontal lines, median values; dashed lines, mean values; vertical lines, data spread up to 1.5 × the interquartile range; closed circles, individual metagenome species richness values; different lower-case letters indicate statistically significant differences (α < 0.05).

Although a single symbiont can provide the complete core set of CAZymes needed by each host individual, most hosts include two to five symbiont species in their gill communities. This observation suggests that hosts might increase the diversity and flexibility of their wood-digestion toolkits by recruiting additional symbionts to their gill communities. By analyzing the diversity of lignocellulose-active CAZyme module subfamilies in all 36 gill metagenomes from *T. bartschi* and *L. pedicellatus*, we found that, on average, the number of unique lignocellulose-active CAZyme subfamilies increases significantly (Kruskal–Wallis test, α < 0.05) as the number of symbionts per individual rises from one to three (Dunn’s test with Holm correction, α < 0.05). However, recruiting more symbionts does not lead to further significant increases (Fig. 8). Therefore, it may not be coincidental that the most common patterns of symbiont occurrence in both host species include three symbiont species (Fig. 5). This level of species richness may optimize CAZyme diversity relative to the costs of acquiring and maintaining additional symbionts.

## Summary and conclusions

Our results demonstrate that these two host species share multiple symbiont species, and that the abilities to fix nitrogen and to degrade all major lignocellulose components are common to all symbiont communities examined. Thus, although these symbiont communities have interchangeable elements, their composition is neither random nor determined by host phylogenetic identity; instead, it is shaped by the individual metabolic needs of the hosts and symbionts, as well as the interactions that occur among them.

## Supplementary Material

Supplementary_Figure_S1_revised_wrag089

Supplementary_Table_S1_2026-04-09_wrag089

Supplementary_Table_S2_2026-02-12_wrag089

Suplementary_Table_S3_2025-09-30_wrag089

## Data Availability

The datasets generated and analyzed in the current study are included in this published article and its supplementary information files (http://dx.doi.org/10.5061/dryad.ksn02v7jd). Genome sequence data for MAGs and isolates are available in the NCBI Genbank repository, under NCBI Bioproject PRJNA1308587 and accession numbers SAMN51758144 (PMS-3907K.s.1b.02), and SAMN50737058 (Lp-A-06).
