# Supplementary figures and images for: Metabolic potential structures gill symbiont communities in two common shipworm species
Source: ISME J. 2026 Apr 23;20(1):wrag089. doi: 10.1093/ismejo/wrag089 (PMC13140551; doi:10.1093/ismejo/wrag089)

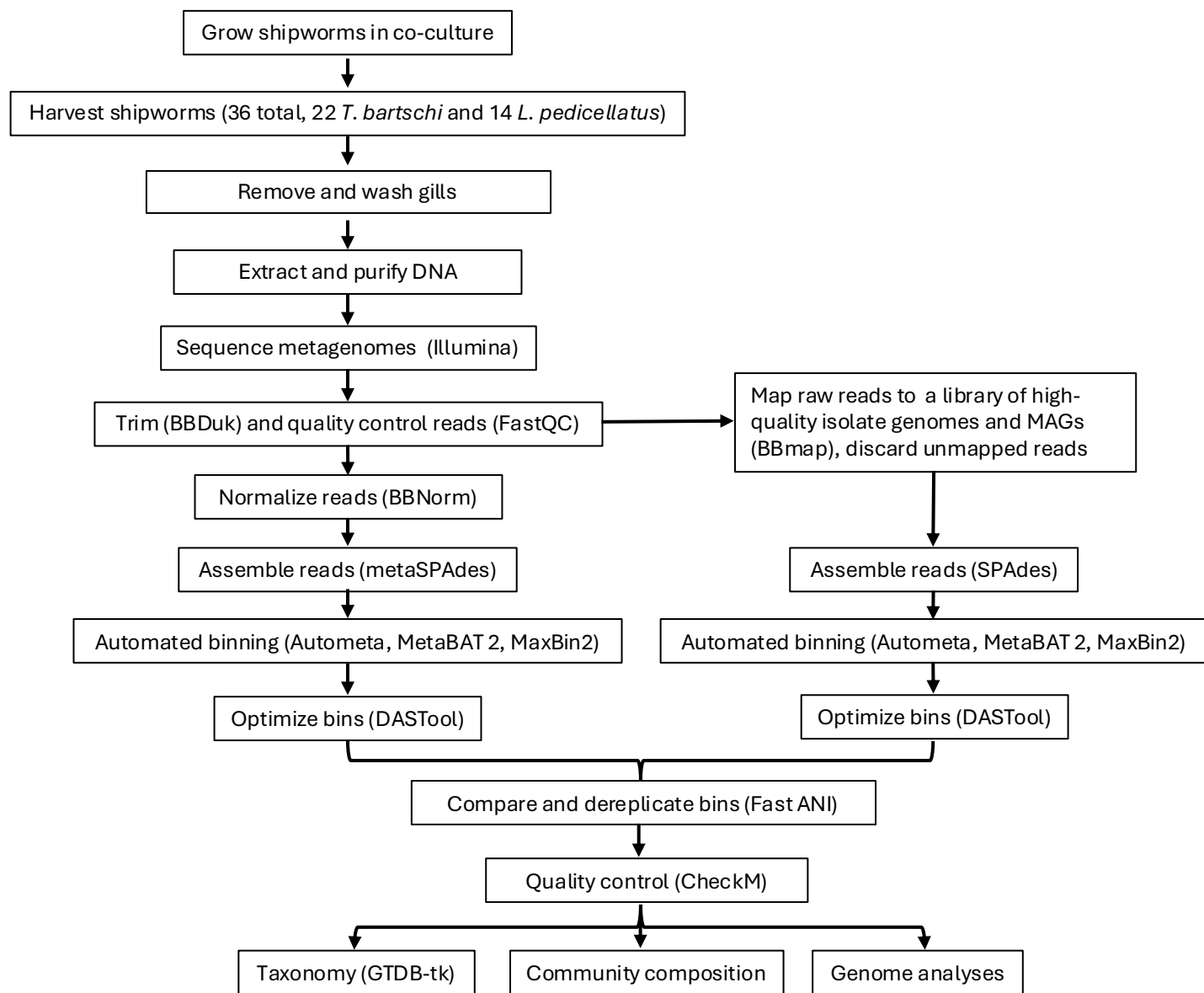

Supplement: Supplementary_Figure_S1_revised_wrag089 [file supplementary_figure_s1_revised_wrag089.pdf]
