# Supplementary material for: Metabolic potential structures gill symbiont communities in two common shipworm species
Source: ISME J. 2026 Apr 23;20(1):wrag089. doi: 10.1093/ismejo/wrag089 (PMC13140551; doi:10.1093/ismejo/wrag089)
Supplement: Supplementary_Table_S1_2026-04-09_wrag089 [file supplementary_table_s1_2026-04-09_wrag089.docx]

Supplementary Table S1. List of specimens used in this study, including sample ID, host species name, and sample processing date.

| Sample ID | Host species | Processing date (yyyy-mm-dd) |
| --- | --- | --- |
| RF-152 | *Teredo bartschi* | 2023-08-28 |
| RF-157 | *Lyrodus pedicellatus* | 2023-08-28 |
| RF-151 | *Teredo bartschi* | 2023-08-28 |
| RF-154 | *Teredo bartschi* | 2023-08-28 |
| RF-158 | *Teredo bartschi* | 2023-08-28 |
| RF-149 | *Lyrodus pedicellatus* | 2023-08-28 |
| RF-153 | *Lyrodus pedicellatus* | 2023-08-28 |
| RF-156 | *Lyrodus pedicellatus* | 2023-08-28 |
| RF-150 | *Teredo bartschi* | 2023-08-28 |
| RF-155 | *Teredo bartschi* | 2023-08-28 |
| RF-214 | *Teredo bartschi* | 2024-05-21 |
| RF-227 | *Lyrodus pedicellatus* | 2025-01-09 |
| RF-228 | *Lyrodus pedicellatus* | 2025-01-09 |
| RF-229 | *Lyrodus pedicellatus* | 2025-01-09 |
| RF-230 | *Lyrodus pedicellatus* | 2025-01-09 |
| RF-231 | *Lyrodus pedicellatus* | 2025-01-09 |
| RF-232 | *Teredo bartschi* | 2025-01-09 |
| RF-233 | *Teredo bartschi* | 2025-01-09 |
| RF-234 | *Teredo bartschi* | 2025-01-09 |
| RF-235 | *Teredo bartschi* | 2025-01-09 |
| RF-236 | *Teredo bartschi* | 2025-01-09 |
| RF-237 | *Teredo bartschi* | 2025-01-10 |
| RF-238 | *Teredo bartschi* | 2025-01-10 |
| RF-239 | *Teredo bartschi* | 2025-01-10 |
| RF-240 | *Teredo bartschi* | 2025-01-10 |
| RF-241 | *Teredo bartschi* | 2025-01-10 |
| RF-242 | *Teredo bartschi* | 2025-01-10 |
| RF-243 | *Teredo bartschi* | 2025-01-10 |
| RF-245 | *Lyrodus pedicellatus* | 2025-01-13 |
| RF-246 | *Lyrodus pedicellatus* | 2025-01-13 |
| RF-247 | *Teredo bartschi* | 2025-01-13 |
| RF-251 | *Teredo bartschi* | 2025-01-13 |
| RF-252 | *Teredo bartschi* | 2025-01-13 |
| RF-248 | *Lyrodus pedicellatus* | 2025-01-14 |
| RF-249 | *Lyrodus pedicellatus* | 2025-01-14 |
| RF-250 | *Lyrodus pedicellatus* | 2025-01-14 |
