# Supplementary material for: Metabolic potential structures gill symbiont communities in two common shipworm species
Source: ISME J. 2026 Apr 23;20(1):wrag089. doi: 10.1093/ismejo/wrag089 (PMC13140551; doi:10.1093/ismejo/wrag089)
Supplement: Supplementary_Table_S2_2026-02-12_wrag089 [file supplementary_table_s2_2026-02-12_wrag089.docx]

Supplementary Table S2. Identification of host species based on pairwise identity of the recovered portion of the mitochondrial genome and the mitochondrial cytochrome oxidase (COI) gene from each sample.

| Sample ID | Morphological identification | Comparison to GenBank reference mitogenome: | | Comparison to GenBank reference COI: | |
| --- | --- | --- | --- | --- | --- |
|  |  | *L. pedicellatus* (OM910820) | *T. bartschi* (OM910823) | *L. pedicellatus* (OM910820) | *T. bartschi* (OM910823) |
|  |  | Identity (%) / recovered portion (%) | Identity (%) / recovered portion (%) | Identity (%) / recovered portion (%) | Identity (%) / recovered portion (%) |
| LP-RF-149 | *Lyrodus pedicellatus* | 99.9/99.8 | 69.5/100 | 99.4/100 | 84.5/100 |
| LP-RF-153 | *Lyrodus pedicellatus* | 99.8/99.8 | 72.7/69.6 | 100/100 | 84.8/100 |
| LP-RF-156 | *Lyrodus pedicellatus* | 99.9/99.8 | 72.6/69.5 | 99.4/100 | 84.5/100 |
| LP-RF-157 | *Lyrodus pedicellatus* | 99.3/99.3 | 62.2/62.5 | 100/100 | 83.3/100 |
| LP-RF-227 | *Lyrodus pedicellatus* | 99.3/100 | 62.5/100 | 100/100 | 83.3/100 |
| LP-RF-228 | *Lyrodus pedicellatus* | 99.3/98.9 | 74.5/99 | 100/100 | 83.3/100 |
| LP-RF-229 | *Lyrodus pedicellatus* | 99.3/100 | 62.1/100 | 100/100 | 83.3/100 |
| LP-RF-230 | *Lyrodus pedicellatus* | 99.3/98.9 | 74.5/98.9 | 100/100 | 83.3/100 |
| LP-RF-231 | *Lyrodus pedicellatus* | 99.3/98.8 | 74.1/99.2 | 100/100 | 83.3/100 |
| LP-RF-245 | *Lyrodus pedicellatus* | 99.3/98.9 | 74.5/98.9 | 100/100 | 83.3/100 |
| LP-RF-246 | *Lyrodus pedicellatus* | 99.3/98.8 | 74.4/98.9 | 100/100 | 83.3/100 |
| LP-RF-248 | *Lyrodus pedicellatus* | 99.3/100 | 62.5/100 | 100/100 | 83.3/100 |
| LP-RF-249 | *Lyrodus pedicellatus* | 99.3/100 | 62.5/100 | 100/100 | 83.3/100 |
| LP-RF-250 | *Lyrodus pedicellatus* | 99.3/100 | 62.1/100 | 100/100 | 83.3/100 |
| TB-RF-150 | *Teredo bartschi* | 68.9/93.2 | 99.1/100 | 83.4/100 | 99.4/100 |
| TB-RF-151 | *Teredo bartschi* | 87.8/96.3 | 99/100 | 89.6/100 | 99/100 |
| TB-RF-152 | *Teredo bartschi* | 81.3/37.1 | 99.4/39.1 | 83.6/71.1 | 100/71.1 |
| TB-RF-154 | *Teredo bartschi* | 74.1/93.3 | 99.7/100 | 83/100 | 99.7/100 |
| TB-RF-155 | *Teredo bartschi* | 90.8/49 | 98.4/100 | 88.4/100 | 98.2/100 |
| TB-RF-158 | *Teredo bartschi* | 92.3/96.1 | 98.5/100 | 89.2/100 | 98.3/100 |
| TB-RF-232 | *Teredo bartschi* | 81.2/59 | 99.9/89 | 83.3/97.6 | 100/97.6 |
| TB-RF-233 | *Teredo bartschi* | 80.3/57.9 | 99.9/87.9 | 83.3/97.6 | 100/97.6 |
| TB-RF-234 | *Teredo bartschi* | 81.2/43.7 | 100/47.6 | 83.3/81 | 100/81 |
| TB-RF-235 | *Teredo bartschi* | 81.7/38.4 | 100/40.5 | 83.6/71.1 | 100/71.1 |
| TB-RF-236 | *Teredo bartschi* | 81.4/38.9 | 100/42.4 | 83.6/71.2 | 100/71.2 |
| TB-RF-237 | *Teredo bartschi* | 81.6/31.1 | 100/32.7 | 83.7/71.2 | 100/71.2 |
| TB-RF-238 | *Teredo bartschi* | 81.6/40.2 | 100/42.4 | 83.7/71.2 | 100/71.2 |
| TB-RF-239 | *Teredo bartschi* | 81.4/33.8 | 97.2/40.4 | 83.7/71.2 | 100/71.2 |
| TB-RF-240 | *Teredo bartschi* | 82.1/33 | 97.1/42.6 | 83.6/81.9 | 100/88.5 |
| TB-RF-241 | *Teredo bartschi* | 81.5/34.2 | 99.8/39.3 | 84.2/55.2 | 99.8/78.4 |
| TB-RF-242 | *Teredo bartschi* | 81.6/35.7 | 100/37.6 | 83.6/71.2 | 100/71.2 |
| TB-RF-243 | *Teredo bartschi* | 81.9/33.3 | 100/35.1 | 83.6/71 | 100/71 |
| TB-RF-247 | *Teredo bartschi* | 80/66.4 | 99.9/88.9 | 83.3/97.5 | 100/97.5 |
| TB-RF-251 | *Teredo bartschi* | 79.5/72.1 | 99.9/89.7 | 83.3/97.5 | 100/97.5 |
| TB-RF-252 | *Teredo bartschi* | 80.6/66.2 | 99.9/90 | 83.3/97.6 | 100/97.6 |
| TB-RF-214 | *Teredo bartschi* | 81.7/37 | 100/39 | 83.6/70.9 | 100/70.9 |
