# Supplementary material for: Metabolic potential structures gill symbiont communities in two common shipworm species
Source: ISME J. 2026 Apr 23;20(1):wrag089. doi: 10.1093/ismejo/wrag089 (PMC13140551; doi:10.1093/ismejo/wrag089)
Supplement: Suplementary_Table_S3_2025-09-30_wrag089 [file suplementary_table_s3_2025-09-30_wrag089.docx]

**Supplementary Table S3. Core Lignocellulose-active CAZyme module subfamilies and their predicted substrates and activities.** CAZy, Carbohydrate-active Enzyme Database, <https://www.cazy.org>; BRENDA Enzyme Database, <https://www.brenda-enzymes.info/index.php>.

| Subfamily name | Predicted substrate | Lignocellulose component | Predicted activity (EC) | Notes | Reference source |
| --- | --- | --- | --- | --- | --- |
| AA2_e1 | lignin | lignin | EC 1.11.1.14 - lignin peroxidase | Lignin-modifying peroxidase | CAZy |
| CBM22_e11 | [xylan, β-glucan](https://bcb.unl.edu/dbCAN2/download/dbsub_data/Substrate_05-03-2022.txt) | hemicellulose | NA | Primarily binds xylan, weaker binding to soluble β-1,4-glucans and β-1,3-β1,4-mixed linked glucans, and in one report, crystalline cellulose | CAZy |
| CBM22_e13 | xylan | hemicellulose | NA | Primarily binds xylan, weaker binding to soluble β-1,4-glucans and β1,3-β-1,4-mixed linked glucans, and in one report, crystalline cellulose | CAZy |
| CBM35_e4 | [xylan, β-mannan, β-galactan](https://bcb.unl.edu/dbCAN2/download/dbsub_data/Substrate_05-03-2022.txt) | hemicellulose | NA | Binds to decorated soluble mannans and mannooligosaccharides, and β-galactan. | CAZy |
| CBM59_e3 | cellulose, mannan, xylan | cellulose, hemicellulose | NA | Binding to mannan, xylan, and cellulose | CAZy |
| CBM6_e43 | cellulose, xylan | cellulose, hemicellulose | NA | Binding specificities for linear and branched/decorated xylan, β-1,4-glucan (or cellulose), mixed-linked β-1,3-1,4-glucan | CAZy |
| CE6_e3 | [xylan](https://bcb.unl.edu/dbCAN2/download/dbsub_data/Substrate_05-03-2022.txt) | hemicellulose | EC 3.1.1.72 - acetylxylan esterase | Deacetylation of xylans and xylo-oligosaccharides | dbCAN, BRENDA |
| GH10_e75 | [xylan](https://bcb.unl.edu/dbCAN2/download/dbsub_data/Substrate_05-03-2022.txt) | hemicellulose | EC 3.2.1.8 - endo-1,4-β-xylanase | Endohydrolysis of (1->4)-β-D-xylosidic linkages in xylans | dbCAN, BRENDA |
| GH11_e23 | [xylan](https://bcb.unl.edu/dbCAN2/download/dbsub_data/Substrate_05-03-2022.txt) | hemicellulose | EC 3.2.1.8 - endo-1,4-β-xylanase | Endohydrolysis of (1->4)-β-D-xylosidic linkages in xylans | dbCAN, BRENDA |
| GH115_e0 | [xylan](https://bcb.unl.edu/dbCAN2/download/dbsub_data/Substrate_05-03-2022.txt) | hemicellulose | EC 3.2.1.131 - xylan α-1,2-glucuronosidase | Hydrolysis of (1->2)-α-D-(4-O-methyl)glucuronosyl links in the main chain of hardwood xylans | dbCAN, BRENDA |
| GH130_e6 | [β-mannan](https://bcb.unl.edu/dbCAN2/download/dbsub_data/Substrate_05-03-2022.txt) | hemicellulose | EC 2.4.1.281 - 4-O-β-D-mannosyl-D-glucose phosphorylase | 4-O-β-D-mannopyranosyl-D-glucopyranose + phosphate = D-glucose + α-D-mannose 1-phosphate | dbCAN, BRENDA |
| GH26_e32 | [β-mannan](https://bcb.unl.edu/dbCAN2/download/dbsub_data/Substrate_05-03-2022.txt) | hemicellulose | EC 3.2.1.78 - mannan endo-1,4-β-mannosidase | Random hydrolysis of (1->4)-β-D-mannosidic linkages in mannans, galactomannans and glucomannans | dbCAN, BRENDA |
| GH3_e0 | [β-glucan](https://bcb.unl.edu/dbCAN2/download/dbsub_data/Substrate_05-03-2022.txt) | hemicellulose | EC 3.2.1.21 - β-glucosidase | Wide specificity for β-D-glucosides. Some examples also hydrolyse one or more of the following: β-D-galactosides, α-L-arabinosides, β-D-xylosides, β-D-fucosides. | dbCAN, BRENDA |
|  | [xylan](https://bcb.unl.edu/dbCAN2/download/dbsub_data/Substrate_05-03-2022.txt) | hemicellulose | EC 3.2.1.37 - xylan 1,4-β-xylosidase | Hydrolysis of (1->4)-β-D-xylans, to remove successive D-xylose residues from the non-reducing termini | dbCAN, BRENDA |
|  | [arabinan](https://bcb.unl.edu/dbCAN2/download/dbsub_data/Substrate_05-03-2022.txt) | hemicellulose | EC 3.2.1.55 - non-reducing end α-L-arabinofuranosidase | hydrolysis of terminal non-reducing α-L-arabinofuranoside residues in α-L-arabinosides | dbCAN, BRENDA |
|  | β-glucan | hemicellulose | EC 3.2.1.6 - endo-1,3(4)-β-glucanase | Substrates include laminarin, lichenin and cereal D-glucans | dbCAN, BRENDA |
|  | β-glucan | hemicellulose | EC 3.2.1.73 - licheninase | Acts on lichenin and cereal β-D-glucans, but not on β-D-glucans containing only 1,3- or 1,4-bonds. | dbCAN, BRENDA |
| GH3_e227 | [xyloglucan](https://bcb.unl.edu/dbCAN2/download/dbsub_data/Substrate_05-03-2022.txt) | hemicellulose | EC 3.2.1.155 - xyloglucan-specific endo-processive β-1,4-glucanase | Hydrolysis of (1->4)-D-glucosidic linkages in xyloglucans so as to successively remove oligosaccharides from the chain end. | dbCAN, BRENDA |
|  | [β-glucan](https://bcb.unl.edu/dbCAN2/download/dbsub_data/Substrate_05-03-2022.txt) | hemicellulose | EC 3.2.1.21 - β-glucosidase | Wide specificity for β-D-glucosides. Some examples also hydrolyse one or more of the following: β-D-galactosides, α-L-arabinosides, β-D-xylosides, β-D-fucosides. | dbCAN, BRENDA |
|  | [β-glucan](https://bcb.unl.edu/dbCAN2/download/dbsub_data/Substrate_05-03-2022.txt) | hemicellulose | EC 3.2.1.6 - endo-1,3(4)-β-glucanase | Endohydrolysis of (1->3)- or (1->4)-linkages in β-D-glucans when the glucose residue whose reducing group is involved in the linkage to be hydrolysed is itself substituted at C-3 | dbCAN, BRENDA |
|  | [β-glucan](https://bcb.unl.edu/dbCAN2/download/dbsub_data/Substrate_05-03-2022.txt) | cellulose | EC 3.2.1.74 - glucan 1,4-β-glucosidase | Hydrolysis of (1->4)-linkages in (1->4)-β-D-glucans, to remove successive glucose units, synonyms cellobiohydrolase, exoglucanase, cellobiase, glucohydrolase, carboxymethyl cellulase, | dbCAN, BRENDA |
| GH43_e53 | [xylan](https://bcb.unl.edu/dbCAN2/download/dbsub_data/Substrate_05-03-2022.txt) | hemicellulose | 3.2.1.37 xylan 1,4-β-xylosidase | Hydrolysis of (1->4)-β-D-xylans, to remove successive D-xylose residues from the non-reducing termini | dbCAN, BRENDA |
|  | [xylan](https://bcb.unl.edu/dbCAN2/download/dbsub_data/Substrate_05-03-2022.txt) | hemicellulose | 3.2.1.55 non-reducing end α-L-arabinofuranosidase | Hydrolysis of terminal non-reducing α-L-arabinofuranoside residues in α-L-arabinosides | dbCAN, BRENDA |
| GH5_e22 | β-mannan | hemicellulose | EC 3.2.1.78 - mannan endo-1,4-β-mannosidase | Random hydrolysis of (1->4)-β-D-mannosidic linkages in mannans, galactomannans and glucomannans | dbCAN, BRENDA |
| GH5_e266 | cellulose | cellulose | EC 3.2.1.4 - cellulase | Endohydrolysis of (1->4)-β-D-glucosidic linkages in cellulose, lichenin and cereal β-D-glucans | dbCAN, BRENDA |
|  | [β-glucan](https://bcb.unl.edu/dbCAN2/download/dbsub_data/Substrate_05-03-2022.txt) | hemicellulose | EC 3.2.1.73 - licheninase | Hydrolysis of (1->4)-β-D-glucosidic linkages in β-D-glucans containing (1->3)- and (1->4)-bonds | dbCAN, BRENDA |
| GH5_e64 | [cellulose](https://bcb.unl.edu/dbCAN2/download/dbsub_data/Substrate_05-03-2022.txt) | cellulose | EC 3.2.1.74 - glucan 1,4-β-glucosidase | Hydrolysis of (1->4)-linkages in (1->4)-β-D-glucans, to remove successive glucose units. Acts on 1,4-β-D-glucans and related oligosaccharides. Cellobiose is hydrolysed, but very slowly. | dbCAN, BRENDA |
|  | β-mannan | hemicellulose | EC 3.2.1.78 - mannan endo-1,4-β-mannosidase | Random hydrolysis of (1->4)-β-D-mannosidic linkages in mannans, galactomannans and glucomannans | dbCAN, BRENDA |
| GH5_e7 | [β-glucan](https://bcb.unl.edu/dbCAN2/download/dbsub_data/Substrate_05-03-2022.txt) | cellulose | EC 3.2.1.4 - cellulase | Endohydrolysis of (1->4)-β-D-glucosidic linkages in cellulose, lichenin and cereal β-D-glucans | dbCAN, BRENDA |
|  | [cellulose](https://bcb.unl.edu/dbCAN2/download/dbsub_data/Substrate_05-03-2022.txt) |  | EC 3.2.1.91 - cellulose 1,4-β-cellobiosidase (non-reducing end) | Hydrolysis of (1->4)-β-D-glucosidic linkages in cellulose and cellotetraose, releasing cellobiose from the non-reducing ends of the chains | dbCAN, BRENDA |
| GH6_e0 | [cellulose](https://bcb.unl.edu/dbCAN2/download/dbsub_data/Substrate_05-03-2022.txt) | cellulose | EC 3.2.1.4 - cellulase | Endohydrolysis of (1->4)-β-D-glucosidic linkages in cellulose, lichenin and cereal β-D-glucans | dbCAN, BRENDA |
|  | [cellulose](https://bcb.unl.edu/dbCAN2/download/dbsub_data/Substrate_05-03-2022.txt) | cellulose | EC 3.2.1.91 - cellulose 1,4-β-cellobiosidase (non-reducing end) | Hydrolysis of (1->4)-β-D-glucosidic linkages in cellulose and cellotetraose, releasing cellobiose from the non-reducing ends of the chains | dbCAN, BRENDA |
|  | xylan | hemicellulose | EC 3.2.1.8 - endo-1,4-β-xylanase | Endohydrolysis of (1->4)-β-D-xylosidic linkages in xylans | dbCAN, BRENDA |
| GH8_e19 | xylan | hemicellulose | EC 3.2.1.32 - endo-1,3-β-xylanase | Random endohydrolysis of (1->3)-β-D-glycosidic linkages in (1->3)-β-D-xylans. The enzyme produces mainly xylobiose, xylotriose and xylotetraose | dbCAN, BRENDA |
|  | [β-glucan](https://bcb.unl.edu/dbCAN2/download/dbsub_data/Substrate_05-03-2022.txt) | hemicellulose | EC 3.2.1.6 - endo-1,3(4)-β-glucanase | Endohydrolysis of (1->3)- or (1->4)-linkages in β-D-glucans when the glucose residue whose reducing group is involved in the linkage to be hydrolysed is itself substituted at C-3 | dbCAN, BRENDA |
|  | xylan | hemicellulose | EC 3.2.1.8 - endo-1,4-β-xylanase | Endohydrolysis of (1->4)-β-D-xylosidic linkages in xylans | dbCAN, BRENDA |
| GH9_e1 | [cellulose](https://bcb.unl.edu/dbCAN2/download/dbsub_data/Substrate_05-03-2022.txt) | cellulose | EC 3.2.1.4 - cellulase | Endohydrolysis of (1->4)-β-D-glucosidic linkages in cellulose, lichenin and cereal β-D-glucans | dbCAN, BRENDA |
| GH9_e26 | [cellulose](https://bcb.unl.edu/dbCAN2/download/dbsub_data/Substrate_05-03-2022.txt) | cellulose | EC 3.2.1.4 - cellulase | Endohydrolysis of (1->4)-β-D-glucosidic linkages in cellulose, lichenin and cereal β-D-glucans | dbCAN, BRENDA |
|  | β-glucan, xyloglucan | hemicellulose | EC 3.2.1.151 - xyloglucan-specific endo-β-1,4-glucanase | Endohydrolysis of 1,4-β-D-glucosidic linkages in xyloglucan | dbCAN, BRENDA |
